# Development and Application of SSR Markers for the QTL *qPHS8-1* Associated with Pre-Harvest Sprouting in Tartary Buckwheat

**DOI:** 10.3390/ijms27135924

**Published:** 2026-06-30

**Authors:** Guohong Tang, Taoxiong Shi, Hongyou Li, Fang Cai, Qingfu Chen, Liwei Zhu

**Affiliations:** 1 Research Center of Buckwheat Industry Technology, College of Life Science, Guizhou Normal University, Guiyang 550025, China; 232100100403@gznu.edu.cn (G.T.); shitaoxiong@gznu.edu.cn (T.S.); 13167515928@163.com (H.L.); caifang919@gmail.com (F.C.); cqf1966@163.com (Q.C.); 2 Guizhou Key Laboratory of Biology and Breeding for Specialty Crops, Guiyang 550006, China

**Keywords:** pre-harvest sprouting, SSR, MAS, genetic diversity

## Abstract

Pre-harvest sprouting (PHS) is a major agronomic problem that limits the yield and quality of Tartary buckwheat. Developing molecular markers closely linked to PHS resistance is important for breeding. In this study, a population of 218 XJ-RILs derived from a cross between the PHS-susceptible cultivar ‘Xiaomiqiao’ and the PHS-resistant cultivar ‘Jinqiaomai 2’ was used as the experimental material. Within the physical interval of the PHS-related QTL locus *qPHS8-1* (8,530,000–9,650,000 bp) in Tartary buckwheat, SSR locus mining, primer design, and polymorphism screening were performed, followed by correlation analysis combining germination phenotypes with molecular markers. A total of 218 SSR loci were identified within this interval, mainly consisting of di-nucleotide (42.66%) and mono-nucleotide (31.19%) repeats. Short repeat motifs were predominantly composed of A/T and AT/AT. Among 114 primer pairs, five polymorphic SSR primers (PHS-8, PHS-23, PHS-29, PHS-65, and PHS-89) were selected. Association analysis showed that lines carrying the maternal allele exhibited significantly higher germination percentage, germination energy, and germination index than those carrying the paternal allele (*p* < 0.01). Cluster analysis further divided the population into four genetic groups, showing clear parental background differentiation consistent with the PHS phenotypes. The five SSR markers developed in this study can be used for marker-assisted selection for PHS resistance in Tartary buckwheat and provide a molecular basis for elucidating the genetic regulatory mechanisms underlying this trait.

## 1. Introduction

Tartary buckwheat (*Fagopyrum tataricum*) is an important medicinal and edible crop. It is rich in flavonoids, such as rutin and quercetin, as well as high-quality protein and dietary fiber, which contribute to regulating human metabolism and preventing cardiovascular diseases [1,2,3]. As a characteristic economic crop in the agricultural regions of southwestern China [4], it holds a crucial traditional position in both production and consumption nationwide. China ranks among the leading countries globally in terms of cultivation area, total yield, and export volume [5]. The Tartary buckwheat industry plays a vital role in ensuring food security and promoting economic development.

However, climate change in recent years has led to the frequent occurrence of pre-harvest sprouting (PHS) in major Tartary buckwheat-producing regions [6], which has become a major constraint on yield and quality stability. PHS refers to the physiological process in which grains germinate on the panicle while still attached to the parent plant. This process typically occurs from grain filling to maturation [7]. High temperature and humidity caused by precipitation during the harvest period can directly trigger germination metabolism in grains before harvest [8,9]. This results not only in direct yield losses but also in the deterioration of the nutritional and processing quality of the grains. Extensive studies have examined the effects of PHS. In their study on the effects of pre-harvest sprouting on the morphological structure and physicochemical properties of rice starch, Zhu et al. concluded that pre-harvest sprouting leads to a significant decrease in grain processing quality and protein content [10]. Park et al. simulated rainy conditions through artificial rainfall and found that, during the critical maturation period of wheat, simulated rainfall increased α-amylase activity and decreased the falling number of grains, leading to deterioration of dough quality [11]. Additionally, germination reduces the amylose and total starch contents in rice and negatively affects its eating quality [12].

Meteorological conditions are key external factors inducing PHS, whereas resistance is essentially determined by the genetic makeup of the variety itself. Therefore, breeding PHS-resistant Tartary buckwheat varieties is a fundamental solution to this problem. PHS resistance is a complex quantitative trait controlled by multiple genes and highly susceptible to environmental fluctuations [13,14,15]. Conventional resistance breeding relies primarily on phenotypic selection. However, this approach suffers from several limitations, including long breeding cycles, environmental interference during phenotyping, and low selection efficiency, all of which severely constrain breeding progress [16,17]. Marker-assisted selection (MAS) enables early and precise identification of desirable traits, independent of phenotypic expression. This approach can substantially accelerate breeding efficiency and has become an important tool for crop genetic improvement [18,19]. Simple sequence repeat (SSR) markers are characterized by codominant inheritance, high polymorphism, operational simplicity, cost-effectiveness, and strong stability. They are widely used for cultivar identification and molecular marker-assisted breeding [20,21]. In crops such as wheat [22,23], oilseed rape [24], and sesame [25], multiple SSR markers associated with seed germination or dormancy have been applied in breeding practices. For instance, Xgwm341 and Xbarc170 are tightly linked to quantitative trait loci for PHS in wheat [22], and markers including Xbarc57, Xbarc294, Xbarc310, and Xbarc321 are significantly associated with seed dormancy [26]. These markers provide core technical support for breeding stress-resistant crop varieties.

Compared with major cereal crops, studies on the genetic mechanisms of PHS in Tartary buckwheat started relatively late, and the development of relevant molecular markers still lags behind. In recent years, with the completion of the whole-genome sequencing of Tartary buckwheat [27,28], breakthroughs have been achieved in research on PHS in this species. In our previous study, a recombinant inbred line (RIL) population derived from a cross between ‘Xiaomiqiao’ and ‘Jinqiaomai 2’ was employed to map qPHS8-1, a major QTL cluster controlling pre-harvest sprouting in Tartary buckwheat, which was stably identified and localized to the 8.53–9.65 Mbp interval on chromosome Ft8 [6]. This finding provides a key genetic foundation for the subsequent development of molecular markers. However, to date, no tightly linked SSR markers suitable for breeding practices have been developed for this locus, which greatly restricts the progress of molecular breeding for PHS resistance in Tartary buckwheat. Accordingly, in this study, 218 XJ-RILs derived from a cross between ‘Xiaomiqiao’ (female parent) and ‘Jinqiaomai 2’ (male parent) were used as the experimental materials. We focused on the physical interval of *qPHS8-1* to perform SSR locus mining and polymorphism screening and identified SSR markers associated with PHS in Tartary buckwheat. This study aimed to develop SSR markers applicable to marker-assisted selection for PHS resistance in Tartary buckwheat, provide molecular evidence for elucidating the genetic regulatory mechanism of PHS, and accelerate the breeding of high-quality Tartary buckwheat varieties resistant to PHS.

## 2. Results

### 2.1. Distribution of SSR Loci Within the QTL Confidence Interval

Table 1 summarizes the distribution of different types of SSR loci within the PHS QTL interval *qPHS8-1* (8,530,000–9,650,000 bp) in Tartary buckwheat, including six SSR types ranging from mono- to hexanucleotide repeats. A total of 218 SSR loci were identified in this interval, with a combined length of 5509 bp. Among the repeat motif types, di-nucleotide repeats were the most abundant, with 93 loci accounting for 42.66% of the total SSRs. They also showed the greatest total length (3348 bp) and an average length of 36 bp. Mono-nucleotide repeats were the second-most common, with 68 loci (31.19% of the total SSRs), a total length of 999 bp, and an average length of 14.69 bp. The numbers of tri- to hexanucleotide repeats decreased sequentially, with 30 (13.76%), 21 (9.63%), 4 (1.83%), and 2 (0.92%) loci, respectively. Their average lengths ranged from 17.33 to 24 bp. These results indicate that di-nucleotide and mono-nucleotide repeats are the predominant SSR types within the QTL interval associated with PHS in Tartary buckwheat.

### 2.2. Distribution Characteristics of SSR Motif Abundance

**Figure 1:**
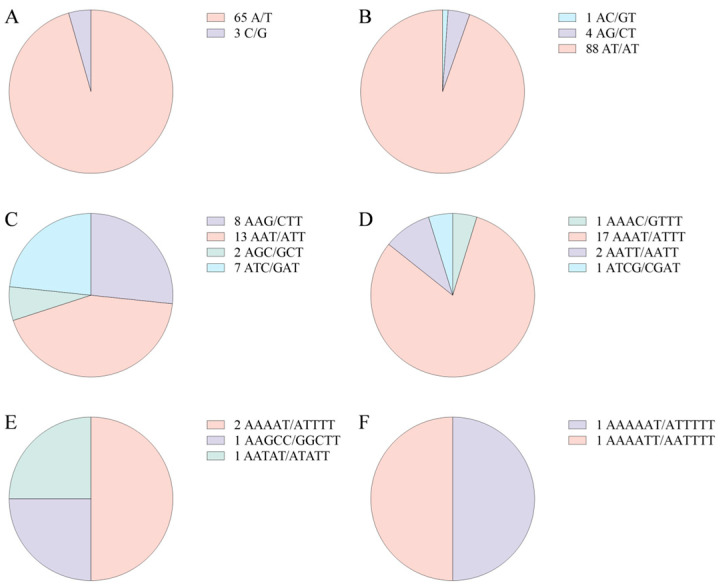
Abundance distribution of SSR motifs in the QTL interval associated with pre-harvest sprouting in Tartary buckwheat. Note: (**A**) Mono-nucleotide; (**B**) Di-nucleotide; (**C**) Tri-nucleotide; (**D**) Tetra-nucleotide; (**E**) Penta-nucleotide; (**F**) Hexa-nucleotide.

Figure 1 illustrates the distribution of repeat motif abundance in SSR loci, ranging from mono- to hexa-nucleotide repeats. In mono-nucleotide SSRs, A/T repeats were the most abundant, with 65 occurrences, whereas C/G repeats were rare (Figure 1A). Di-nucleotide SSRs were predominantly composed of AT/AT repeats, with 88 occurrences. In contrast, AC/GT and AG/CT repeats occurred at extremely low frequencies, with only 1 and 4 occurrences, respectively (Figure 1B). Among tri-nucleotide repeats, AAT/ATT was the most frequent, with 13 occurrences, followed by AAG/CTT, ATC/GAT, and AGC/GCT with 8, 7, and 2 occurrences, respectively, showing a relatively dispersed distribution of abundance (Figure 1C). For tetra-nucleotide SSRs, AAAT/ATTT accounted for a relatively high proportion, with 17 occurrences, although the overall abundance of all motif types remained low (Figure 1D). Penta-nucleotide SSRs contained motifs such as AAAAT/ATTTT, generally showing low and evenly distributed abundance (Figure 1E). Hexa-nucleotide SSRs included only two motif types with equal abundance, each occurring once (Figure 1F). Overall, short-nucleotide SSRs (mono- and di-nucleotide) exhibited dominant repeat motifs, whereas longer nucleotide SSRs (tri- to hexa-nucleotide) displayed greater motif diversity with lower overall abundance. This pattern reflects the distinct distribution of repeat motifs across different SSR lengths.

### 2.3. Distribution Characteristics of SSR Numbers

Table 2 shows the distribution of repeat frequencies for different types of SSRs within the PHS QTL interval in Tartary buckwheat. Among the SSR types, dinucleotide SSRs had the largest number of loci (93) and the widest distribution range of repeat numbers, covering all intervals from 4 to 10 to >50. Mono-nucleotide SSRs contained 68 loci, with repeat numbers mainly concentrated in the 11–20 interval, which included 63 loci. The total numbers of tri- to hexa-nucleotide SSRs decreased as the motif length increased, and their repeat numbers were mainly concentrated in the 4–10 interval. From the overall distribution of repeat number intervals, among the 218 SSR loci, those with repeat numbers of 11–20 accounted for the highest proportion (42.66%), whereas those with repeat numbers greater than 50 represented the lowest proportion (0.92%). These distribution characteristics indicate that, within this QTL interval, short-nucleotide SSRs, particularly dinucleotide SSRs, not only show higher abundance but also exhibit a wider range of repeat number variation. In contrast, long-nucleotide SSRs have relatively conserved repeat numbers and lower abundance.

### 2.4. Polymorphic Primer Screening and Genotyping Analysis

**Figure 2:**
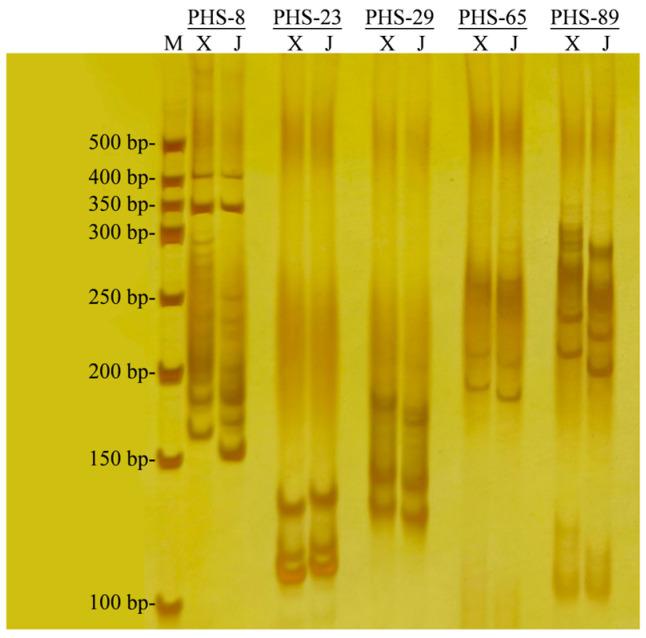
Amplification results of polymorphic SSR markers in the parental lines.

Based on the 218 SSR loci identified within the PHS QTL interval in Tartary buckwheat, a total of 117 primer pairs were designed using Krait v1.1.3 software. After removing primers with identical base sequences in either the forward or reverse strand, 114 primer pairs were synthesized. Among them, 108 primer pairs successfully amplified the target products, and five primer pairs showed polymorphisms between the parents. Detailed information on these polymorphic primers is provided in Table 3, namely PHS-8, PHS-23, PHS-29, PHS-65, and PHS-89. The amplification results for the parental lines are shown in Figure 2.

### 2.5. Association Analysis Between SSR Markers and Germination Traits

**Figure 3:**
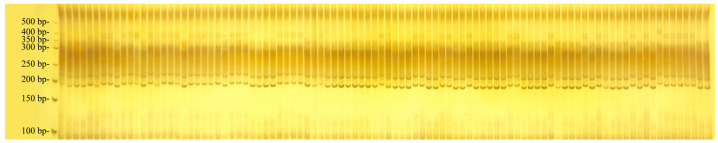
Amplification results of the SSR marker PHS-65 in selected lines of the XJ-RIL population. Note: Electrophoretic lanes from left to right represent the 50 bp DNA ladder, ‘Xiaomiqiao’, ‘Jinqiaomai 2’, and lines R01–R94 of the XJ-RIL population.

Genotyping of the XJ-RIL population was performed using five pairs of polymorphic SSR primers. The genotyping results showed that, for the five SSR markers (PHS-8, PHS-23, PHS-29, PHS-65, and PHS-89), the number of lines exhibiting electrophoretic bands identical to the maternal genotype ‘Xiaomiqiao’ were 102, 100, 99, 97, and 95, respectively. In contrast, the corresponding numbers of lines matching the paternal genotype ‘Jinqiaomai 2’ were 116, 118, 119, 121, and 123, respectively (Figure 3). No heterozygous band type was observed. As shown in Table 4, for the maternal genotype corresponding to the five SSR markers, the germination percentage, germination energy, and germination index of seeds under simulated PHS conditions were extremely significantly higher than those of the corresponding paternal genotype (*p* < 0.01). Although the numbers of maternal and paternal genotype lines varied among markers, the maternal genotype consistently demonstrated dominant performance in germination traits. These results indicate that the SSR markers are strongly associated with the PHS phenotype and can be used to screen Tartary buckwheat germplasm with different levels of PHS.

### 2.6. Cluster Analysis Based on SSR Markers

**Figure 4:**
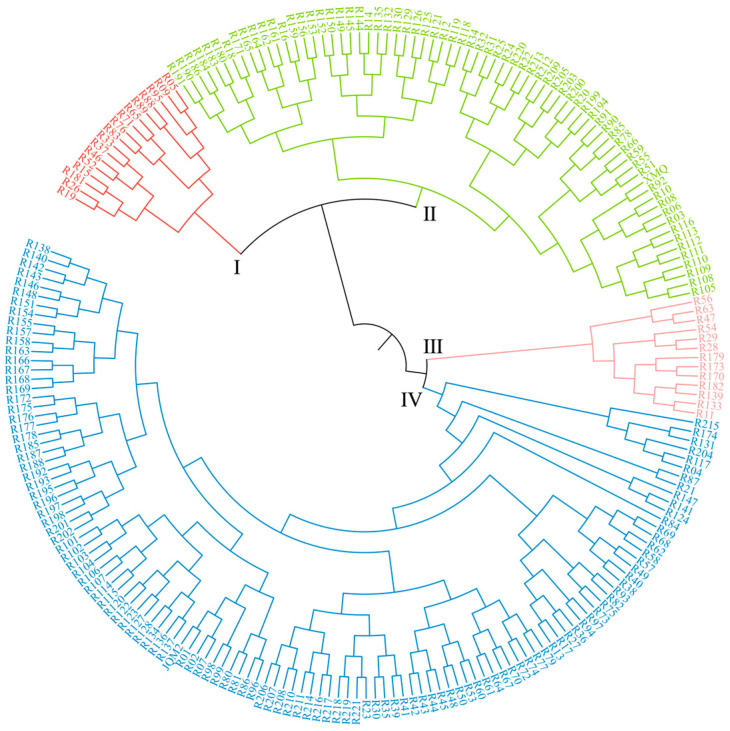
Cluster analysis of the XJ-RIL population based on SSR markers.

The clustering results of the Tartary buckwheat parents and their progeny based on polymorphic SSR markers are presented in Figure 4. The phylogenetic tree divided the parents and the XJ-RIL population into four main genetic clusters. Cluster I contained 17 materials, accounting for 7.73% of the tested materials. This group exhibited the strongest germination ability under simulated PHS conditions, corresponding to the highest PHS percentage. Cluster II included 70 materials, accounting for 31.82% of the tested materials, and the female parent ‘Xiaomiqiao’ (XMQ) was grouped into this cluster. Cluster III contained the fewest materials (13), accounting for only 5.91% of the tested materials. This group showed moderate grain germination ability and the second-highest PHS rate. Cluster IV contained the largest number of materials, accounting for 54.55% of the tested materials, and the male parent ‘Jinqiaomai 2’ (JQM2) was grouped into this cluster. This group showed the weakest germination performance under simulated PHS conditions, corresponding to the lowest PHS percentage, and represented a highly PHS-resistant group.

Analysis of germination differences based on the clustering results in Table 5 showed that the germination percentage, germination energy, and germination index of materials in Cluster I, Cluster II, and Cluster III were significantly higher than those in Cluster IV. In summary, the PHS-susceptible parent ‘Xiaomiqiao’ and the PHS-resistant parent ‘Jinqiaomai 2’ belonged to different clusters with significant differences in their genetic backgrounds. They exhibited a large branch span in the phylogenetic tree. The clustering results not only confirmed the high genetic differentiation between the two parents but also corresponded to the phenotypic differences in their seed germination characteristics.

## 3. Discussion

The distribution of genomic SSRs is non-random. Tri-nucleotide repeats are dominant in coding regions to avoid frameshift mutations, whereas mono-nucleotide and di-nucleotide repeats are more common in UTRs and introns [21]. In this study, a total of 218 SSR loci were identified within the interval of the major PHS QTL *qPHS8-1* in Tartary buckwheat. The distribution pattern showed that di-nucleotide repeats accounted for 42.66%, mono-nucleotide repeats accounted for 31.19%, and tri-nucleotide repeats accounted for 13.76%. This characteristic was slightly different from the SSR types and distribution patterns identified in the whole genome of Tartary buckwheat [29,30] and in the thin-hull gene mapping interval of Tartary buckwheat [31]. This difference is speculated to be related to the parameter settings used during SSR locus scanning and differences in the selection of reference genomes. In addition, this study only scanned part of the gene sequences within the mapping interval, which resulted in incomplete locus identification. However, both this study and previous studies have generally shown that short-chain repeat motifs have a universal advantage in SSR composition. In other crops, for example, SSR loci identified in the whole-genome sequence of potato also showed that mono-nucleotide repeats were the main type, accounting for 62.05% of the total SSRs, followed by di-nucleotide and tri-nucleotide repeats [32]. Fang et al. identified a total of 37,572 SSR loci in the Tartary buckwheat genome, the majority of which were dinucleotide repeats (26, 196, accounting for 69.72%), with the AT/TA repeat motif being the most abundant (24, 742, constituting 65.85%) [33]. In the gene sequences of maize, trinucleotide and hexanucleotide motifs are more abundant in CDS, whereas certain mononucleotide and dinucleotide motifs are more abundant in UTRs [34]. In addition, SSR loci identified in the whole genome of Dendrobium officinale also showed that short-nucleotide repeats were the most abundant [35].

Some scholars have suggested that A/T-rich repeat motifs are more likely to cause DNA strand unwinding and replication slippage, thereby generating higher polymorphism [21]. In the genome, short-sequence SSRs exhibit a fast mutation rate, whereas long-sequence SSRs have a slower mutation rate and are relatively stable. Short-chain SSRs within gene sequences may increase gene instability, but this instability can also drive gene mutation [36,37]. In this study, in terms of repeat motif composition, A/T (65/68) and AT/AT (88/93) repeat motifs were the most abundant. This characteristic is consistent with the general distribution pattern of SSR motifs in the whole genome of Tartary buckwheat reported in previous studies [38]. It suggests that their high polymorphism may have a potential molecular association with the regulation of PHS traits in Tartary buckwheat.

SSR markers developed based on phenotypic traits provide technical support for crop genetic improvement by directly associating target agronomic traits with molecular genotypes. Hou et al. developed the SSR marker SXAU4308, which had a significant effect on the 100-grain weight of Tartary buckwheat in multiple environments [39]. This marker was regarded as an important locus for marker-assisted breeding and yield improvement research. Cheng et al. used 15 SSR markers to evaluate the genetic diversity and population structure of 659 Tartary buckwheat varieties. They identified a core germplasm resource containing 165 germplasms, providing valuable resources for the conservation of genetic diversity and the genetic improvement of Tartary buckwheat [40]. Balážová et al. analyzed the genotypes of 35 buckwheat accessions using 21 pairs of SSR primers and confirmed that SSR molecular marker technology is a suitable tool for identifying interspecific and intraspecific genetic variation and analyzing genetic diversity [41]. Compared with SSR markers associated with PHS in other crops, the five polymorphic markers obtained in this study have a clearer QTL mapping background, which reduces the risk of false positives in marker-assisted selection. Association analysis showed that the germination percentage, germination energy, and germination index of lines carrying alleles from the PHS-susceptible female parent were extremely significantly higher than those of lines carrying alleles from the PHS-resistant male parent. This correlation was consistently observed across all five markers. These results not only verified the major regulatory role of the *qPHS8-1* locus in PHS traits of Tartary buckwheat [6] but also demonstrated that the developed markers could accurately distinguish between resistant and susceptible genotypes. In breeding practice, these markers can be used for the rapid screening of early-generation materials without waiting for phenotypic identification. This approach can significantly shorten the breeding cycle and is particularly suitable for regions with frequent rainfall and strong environmental interference in PHS identification, such as those in Southwest China.

Meanwhile, based on the genetic clustering of the five SSR markers and the PHS phenotype of Tartary buckwheat, this study further clarified the relationship between genetic groups and PHS resistance. The results indicated that the XJ-RIL population exhibited significant genetic differentiation and rich genetic diversity. There were no significant differences in GP, GE, and GI among Cluster I, Cluster II, and Cluster III, whereas all three clusters showed significantly higher germination phenotypes than Cluster IV. These results suggest that the tested population could essentially be divided into two major groups in terms of PHS phenotype. One group was the resistant population represented by Cluster IV, and the other was the PHS-susceptible population composed of Cluster I, Cluster II, and Cluster III. However, previous studies by our research group did not identify a stable correlation between GP, GE, GI, and the PHS rate [6]. The main reason for this difference may be that fresh, undried seeds were used as the experimental materials in this study to simulate seed PHS. This approach maximally restored the physiological state of natural PHS in the field and avoided artificial changes in grain moisture, cell structure, and other factors caused by drying treatments. The simulation conditions were highly consistent with the environmental conditions under which natural PHS occurs in the fields. Therefore, the experimental results could more accurately reflect the field PHS resistance of the tested materials. Similarly, many researchers have used fresh plant materials to evaluate PHS resistance in crops. For example, in crops such as rice [42] and wheat [43], simulated PHS experiments have been conducted using fresh seeds or fresh whole panicles. This approach ensures the authenticity and reliability of PHS resistance evaluation and provides a scientific reference for the assessment, genetic analysis, and germplasm screening of crop PHS resistance.

## 4. Materials and Methods

### 4.1. Plant Materials

The experimental materials were obtained from an XJ-RIL population consisting of 218 lines developed from a cross between ‘Xiaomiqiao’, a thin-shelled and PHS-susceptible Tartary buckwheat cultivar used as the female parent, and ‘Jinqiaomai 2’, a thick-shelled and PHS-resistant cultivar used as the male parent [44]. All materials were planted at the Anshun Experimental Base of the Research Center of Buckwheat Industry Technology, Guizhou Normal University (106°18′ E, 26°17′ N), in August 2024. Individual plants were tagged 30 days after sowing and used for DNA extraction and evaluation of PHS-related traits.

### 4.2. Evaluation of Pre-Harvest Sprouting Traits in Tartary Buckwheat

For each tagged line, 50 fresh, mature, plump, and uniform Tartary buckwheat seeds were selected [45], rinsed with distilled water, and placed in germination boxes for a simulated PHS assay. The artificial climate incubator was set at 25 °C with a photoperiod of 16 h light/8 h dark. Water was added daily to simulate humid and rainy conditions, with three biological replicates. The number of germinated seeds was recorded daily. Germination energy (GE) was calculated on the fifth day, and germination percentage (GP) and germination index (GI) were determined on the seventh day.

### 4.3. DNA Extraction and Quality Assessment

Young leaves were collected from the XJ-RIL population and the parental lines of Tartary buckwheat. Genomic DNA was extracted using the CTAB method [46]. DNA concentration was measured using a micro-ultraviolet spectrophotometer, and DNA quality was assessed using 1% agarose gel electrophoresis. The DNA samples were diluted to 50 ng/μL and stored at −20 °C for subsequent use.

### 4.4. SSR Locus Identification and Primer Design

Based on the QTL interval associated with PHS in Tartary buckwheat using the Pinku1 reference genome [28], SSR loci within the physical interval were identified, and primers were designed using Krait v1.1.3 software. The search criteria were set as follows: repeat numbers of 12, 7, and 5 for mono-, di-, and trinucleotide motifs, respectively, and 4 for tetra-, penta-, and hexanucleotide motifs. The primer design parameters were as follows: primer length of 18–27 bp, product size of 100–300 bp, annealing temperature of 58–65 °C, and GC content of 30–60%. The designed primers were synthesized by General Biosystems (Anhui) Co., Ltd. (Chuzhou, China). and purified by PAGE after synthesis.

### 4.5. PCR Amplification and Polymorphic Primer Screening

To evaluate the availability and polymorphism of the SSR primer pairs, 114 synthesized primer pairs were first used to amplify parental DNA. The PCR products were then subjected to polyacrylamide gel electrophoresis to screen for polymorphic primers. The polymorphic primers were subsequently used to amplify DNA from the XJ-RIL population for genotyping analysis. The SSR-PCR amplification system consisted of 1 μL DNA template, 6 μL of Green Taq Mix, 1 μL of each forward and reverse primers (10 μmol/μL), and 3 μL of ddH_2_O. The PCR program was set as follows: pre-denaturation at 94 °C for 3 min; 35 cycles of denaturation at 94 °C for 15 s, annealing at the optimal temperature for 15 s, and extension at 72 °C for 1 min; followed by a final extension at 72 °C for 5 min.

### 4.6. Denaturing Polyacrylamide Gel Electrophoresis (8%)

PCR products were detected using 8% denaturing polyacrylamide gel electrophoresis, as described by Lv [46]. After electrophoresis, the gels were stained and visualized, and the bands were scored. Electrophoretic band patterns in the XJ-RIL population identical to those of the maternal genotype ‘Xiaomiqiao’ were scored as ‘0’, whereas those identical to the paternal genotype ‘Jinqiaomai 2’ were scored as ‘1’.

### 4.7. Statistical Analysis and Data Processing

An independent-samples *t*-test was performed on the germination percentage, germination energy, germination index, and band-scoring results of the population using IBM SPSS Statistics 27 software. Based on the five SSR markers, cluster analysis was conducted using the unweighted pair-group method with arithmetic mean (UPGMA) in PowerMarker 3.5 software, and a circular phylogenetic tree was constructed. The online tool iTOL (https://itol.embl.de/) was used to optimize the visualization of the cluster tree. Data visualization analysis was performed using GraphPad Prism 10.1.2.

## 5. Conclusions

The five SSR markers developed in this study can be directly applied to marker-assisted selection for PHS resistance in Tartary buckwheat, providing a key tool for shortening the breeding cycle and improving breeding efficiency. Meanwhile, this study clarified the distribution pattern of SSR loci within the PHS QTL *qPHS8-1* interval and the genetic mapping of the target trait. These findings provide an important molecular foundation for subsequent fine mapping, candidate gene cloning, and analysis of the genetic regulatory mechanisms underlying PHS traits in Tartary buckwheat.

## Figures and Tables

**Table 1:** Number and distribution of SSR motif repeats in the QTL interval associated with pre-harvest sprouting in Tartary buckwheat.

Type of SSRs	Counts	Percentage (%)	Total Length (bp)	Average Length (bp)
Mono-nucleotide	68	31.19	999	14.69
Di-nucleotide	93	42.66	3348	36
Tri-nucleotide	30	13.76	660	22
Tetra-nucleotide	21	9.63	364	17.33
Penta-nucleotide	4	1.83	90	22.5
Hexa-nucleotide	2	0.92	48	24
Total	218	100	5509	

**Table 2:** Distribution of SSR numbers for different motif types in the QTL interval associated with pre-harvest sprouting of Tartary buckwheat.

Table of SSRs	Number of Replications						Total
	4–10	11–20	21–30	31–40	41–50	>50	
Mono-nucleotide	0	63	4	1	0	0	68
Di-nucleotide	43	18	15	10	4	3	93
Tri-nucleotide	24	5	1	0	0	0	30
Tetra-nucleotide	21	0	0	0	0	0	21
Penta-nucleotide	4	0	0	0	0	0	4
Hexa-nucleotide	2	0	0	0	0	0	2
Total	94	86	20	11	4	3	218
Percent (%)	31.19	42.66	13.76	9.63	1.83	0.92	100

**Table 3:** Information on polymorphic primers between the two parents of the XJ-RIL population.

Primer No.	Initial Position (bp)	Primer Sequence (5′ to 3′)	Tm (℃)	Predicted Product Length (bp)
PHS-8	121,964	F: AATTTCCAACAGCCAACGCC	57	154
		R: AGACATCTTATTGGATAAGTAGGACG		
PHS-23	310,352	F: GCCGTCGTCCTACAAGTTGG	57	106
		R: TTCCTACCGGATCCTGACCC		
PHS-29	395,259	F: AGCAACATGTTATATAGGTCCAAATGG	57	121
		R: ACCTTAAAATTTGACATGTGGGC		
PHS-65	666,545	F: TCATGGGTTGGGTTAAACTAAACC	56	173
		R: GTTGAACGATCAAACGGACCC		
PHS-89	895,210	F: ATATGCGTATATTCGTGATAACATTGG	56.5	187
		R: GTCACAACATAACCAGCGCC		

**Table 4:** Independent-samples *t*-test of germination traits for different genotypes of SSR markers.

SSR Marker	Genotype	Number of Lines	Germination Percentage (%)	Germination Energy (%)	Germination Index
PHS-8	Maternal	102	50.20 **	42.38 **	34.40 **
	Paternal	116	28.83	23.74	20.38
PHS-23	Maternal	100	52.34 **	44.06 **	36.40 **
	Paternal	118	27.38	22.63	18.92
PHS-29	Maternal	99	50.50 **	42.74 **	35.09 **
	Paternal	119	29.12	23.91	20.16
PHS-65	Maternal	97	51.18 **	43.67 **	35.57 **
	Paternal	121	28.93	23.48	20.03
PHS-89	Maternal	95	50.26 **	43.11 **	34.87 **
	Paternal	123	30.00	24.24	20.80

Note: ** indicates an extremely significant difference at the 0.01 level.

**Table 5:** Analysis of phenotypic differences based on the cluster analysis results.

Cluster	Germination Percentage (%)	Germination Energy (%)	Germination Index
Cluster I	61.96 a	48.40 a	43.41 a
Cluster II	61.18 a	53.44 a	42.42 a
Cluster III	54.10 a	41.32 a	38.64 a
Cluster IV	22.21 b	18.07 b	14.61 b

Note: Different letters indicate significant differences in the same trait among clustering groups (*p* < 0.05).

## Data Availability

The original contributions presented in this study are included in the article. Further inquiries can be directed to the corresponding author.
